# Molecular Epidemiology of Clinical Infections Caused by *Serratia marcescens* Complex in a Tertiary Care Hospital System: Insights From Whole-Genome Sequencing

**DOI:** 10.1093/infdis/jiaf392

**Published:** 2025-08-19

**Authors:** Adam S Komorowski, Michael G Surette, Laura Rossi, Dominique Tertigas, Mark Gaskin, Shahrokh Shekarriz, Andrew G McArthur, Marek Smieja, Dominik Mertz

**Affiliations:** Department of Pathology and Molecular Medicine, McMaster University, Hamilton, Ontario, Canada; Department of Laboratory Medicine, Hamilton Health Sciences and St Joseph's Healthcare, Hamilton, Ontario, Canada; Department of Health Research Methodology, Evidence, and Impact, Faculty of Health Sciences, McMaster University, Hamilton, Ontario, Canada; Michael G. DeGroote Institute for Infectious Disease Research, McMaster University, Hamilton, Ontario, Canada; Research Institute of St Joseph's Healthcare Hamilton, St. Joseph’s Healthcare, Hamilton, Ontario, Canada; Michael G. DeGroote Institute for Infectious Disease Research, McMaster University, Hamilton, Ontario, Canada; Department of Biochemistry and Biomedical Sciences, Faculty of Health Sciences, McMaster University, Hamilton, Ontario, Canada; Division of Gastroenterology, Department of Medicine, McMaster University, Hamilton, Ontario, Canada; Department of Biochemistry and Biomedical Sciences, Faculty of Health Sciences, McMaster University, Hamilton, Ontario, Canada; Division of Gastroenterology, Department of Medicine, McMaster University, Hamilton, Ontario, Canada; Department of Biochemistry and Biomedical Sciences, Faculty of Health Sciences, McMaster University, Hamilton, Ontario, Canada; Department of Laboratory Medicine, Hamilton Health Sciences and St Joseph's Healthcare, Hamilton, Ontario, Canada; Department of Biochemistry and Biomedical Sciences, Faculty of Health Sciences, McMaster University, Hamilton, Ontario, Canada; Michael G. DeGroote Institute for Infectious Disease Research, McMaster University, Hamilton, Ontario, Canada; Department of Biochemistry and Biomedical Sciences, Faculty of Health Sciences, McMaster University, Hamilton, Ontario, Canada; Department of Pathology and Molecular Medicine, McMaster University, Hamilton, Ontario, Canada; Department of Laboratory Medicine, Hamilton Health Sciences and St Joseph's Healthcare, Hamilton, Ontario, Canada; Department of Health Research Methodology, Evidence, and Impact, Faculty of Health Sciences, McMaster University, Hamilton, Ontario, Canada; Michael G. DeGroote Institute for Infectious Disease Research, McMaster University, Hamilton, Ontario, Canada; Research Institute of St Joseph's Healthcare Hamilton, St. Joseph’s Healthcare, Hamilton, Ontario, Canada; Division of Infectious Diseases, Department of Medicine, McMaster University, Hamilton, Ontario, Canada; Department of Health Research Methodology, Evidence, and Impact, Faculty of Health Sciences, McMaster University, Hamilton, Ontario, Canada; Michael G. DeGroote Institute for Infectious Disease Research, McMaster University, Hamilton, Ontario, Canada; Division of Infectious Diseases, Department of Medicine, McMaster University, Hamilton, Ontario, Canada

**Keywords:** Enterobacterales, *Serratia marcescens*, infection control, whole-genome sequencing, drug resistance

## Abstract

**Background:**

*Serratia marcescens* is an opportunistic AmpC β-lactamase-producing Enterobacterales associated with intensive care unit outbreaks, causing high morbidity and mortality. The spatiotemporal dynamics of *Serratia* species and their implications for hospital infection prevention and control remain understudied.

**Methods:**

We prospectively identified patient culture specimens in a multihospital academic healthcare system from 2022 to 2024. We included first-time isolates of *S. marcescens* identified via culture and confirmed by matrix-assisted laser desorption ionization time-of-flight mass spectrometry (MALDI-TOF MS). Isolates underwent whole-genome sequencing on the Illumina NextSeq 2000 platform. We queried assembled genomes using the Comprehensive Antibiotic Resistance Database to identify resistance genes and predict resistomes. We constructed a maximum-likelihood phylogenetic tree using GTDB-Tk–assigned taxonomies. We identified possible links between patients if there was a spatiotemporal overlap and the average nucleotide identity (ANI) of a sequence pair was > 99.0%. We collected relevant patient characteristics via retrospective chart review and analyzed data using descriptive statistics.

**Results:**

Of 147 identified isolates, we included 125. Phenotypic testing suggested either inducible or derepressed AmpC expression in all isolates. Whole-genome sequencing found species-level discordance with MALDI-TOF MS in 64 (51.2%) isolates, suggesting the presence of multiple members of the recently described *S. marcescens* complex causing hospital- or community-associated infections. Only 1 isolate pair had a spatiotemporal link and ANI > 99.0%.

**Conclusions:**

Between-patient transmission of *S. marcescens* complex outside of outbreaks is likely rare. Current MALDI-TOF MS-based identification methods are insufficient to identify *S. marcescens* complex and laboratory reporting should be modified to report only to the level of the complex.

## Teaser

This study of 125 *Serratia marcescens* complex clinical isolates found between-patient transmission to be unlikely outside of known outbreaks. It showed that MALDI-TOF MS misidentifies *S. marcescens* complex members compared to whole-genome sequencing, which may compromise accuracy in outbreak investigations.

*Serratia* species are opportunistic Gram-negative bacterial pathogens of the order Enterobacterales [1]. Among *Serratia* species, *Serratia marcescens* is particularly well known as a cause of significant morbidity and mortality, particularly in neonatal intensive care unit outbreaks [2, 3]. It is a frequent colonizer of the epidermis, as well as the gastrointestinal, urinary, and respiratory tracts in humans, and has been found to persist on medical equipment and sinks in the hospital environment [4–6]. Recent evidence suggests that *S. marcescens,* while causing most human infections within the genus, is but 1 constituent species of a wider *S. marcescens* complex. *Serratia bockelmannii, S. marcescens* sensu stricto, *Serratia nematodiphila, Serratia nevei,* and *Serratia ureilytica* have been proposed as constituent *S. marcescens* complex members [7, 8].

*S. marcescens* complex species possess an Ambler class C β-lactamase, known as AmpC enzymes, which catalyze most β-lactams, cephalosporins, and β-lactam/β-lactamase inhibitor combinations through serine-mediated hydrolysis of the β-lactam ring [9]. AmpC activity can occur either through chromosomally encoded inducible resistance, stable derepression via mutations in regulatory genes, or constitutive expression on mobile genetic elements such as plasmids [9–11]. Other Enterobacterales such as *Enterobacter cloacae, Klebsiella aerogenes,* or *Citrobacter freundii* are at significantly higher risk of inducible AmpC production, with mutation rates favoring AmpC derepression being 50- to 150-fold higher among these high-risk species than in *S. marcescens* [12]. Inducible chromosomal AmpC expression results from the use of aminopenicillins, cephalosporin, and cephamycin antimicrobials, thus liberating oligopeptide residues during bacterial cell lysis that compete with uridine disphosphate-*N*-acetylmuramic acid to bind AmpR, the negative regulator of AmpC expression [13]. Conformational changes in AmpR caused by oligopeptide binding result in increasing AmpC production, leading to risk of treatment failure with the use of β-lactams, cephalosporins, and β-lactam/β-lactamase inhibitors [9].

Enterobacterales with constitutively expressed, plasmid-borne extended-spectrum β-lactamases (ESBLs) are widely recognized as significant causes of morbidity and mortality that are transmitted in healthcare settings, primarily through person-to-person contact and environmental persistence [14, 15]. Gowns and gloves may be used during contact with the patients and/or their environments for those known to be colonized with ESBLs and enhanced environmental cleaning may be instituted to prevent colonization and subsequent infection of other patients [16]. Such infection prevention and control recommendations are not routinely in place for AmpC-producing Enterobacterales like *S. marcescens* complex. The recent work of Aracil-Gisbert and colleagues suggested, however, that environmental reservoirs may play a role in the endemicity of AmpC-producing *S. marcescens* complex isolates in an intensive care unit environment in Spain [8]. For example, they showed that environmental reservoirs of *S. marcescens* complex can serve as milieux for the propagation of plasmids containing carbapenemase genes [8]. It is conceivable that *S. marcescens* complex may propagate and persist in the wider hospital environment, particularly given its predilection for water reservoirs [17], and contribute to patient infections. In this study, we aimed to assess whether unrecognized transmission of *S. marcescens* complex may be occurring within our hospital system by determining the genomic relatedness of patient specimens that tested positive for *S. marcescens* by conventional clinical microbiological identification techniques.

## METHODS

### Study Design

We prospectively identified clinical specimens with *S. marcescens* from 3 adult tertiary care sites, 2 secondary care sites, and 1 pediatric tertiary care site (a total of 2800 beds) from 1 city in Ontario, Canada, from January to December 2022, and again from January to June 2024. These specimens with *S. marcescens* identified from culture were confirmed using the VITEK matrix-assisted laser desorption ionization time-of-flight mass spectrometer (MALDI-TOF MS, bioMérieux). The time periods in the study represented a convenience sample of consecutive patients. Institutional ethics board approval for this work was obtained from the Hamilton Integrated Research Ethics Board (project No. 13873).

Specimens were included if they were submitted to diagnose active infection; those submitted to the laboratory to diagnose microbial colonization (ie, rectal swabs) for the purposes of infection prevention and outbreak control were excluded from the analysis. Specimens sampled from any anatomical site, from hospital inpatients and outpatients at the eligible hospital sites were included. Only the first *S. marcescens* isolate from each hospital admission was included.

At the end of each prospective specimen identification period (eg, December 2022, July 2024), a retrospective chart review was undertaken to identify patient characteristics and provide critical epidemiologic information. The data extraction process was piloted on the first 5 identified isolates to ensure all relevant variables were being collected. Microbiologic data extracted from the patient chart were as follows: specimen site; specimen collection date; species identified by MALDI TOF-MS; percent certainty of MALDI TOF-MS identification > 95%; minimum inhibitory concentrations (MIC); and interpretative breakpoints for all antimicrobials tested. Epidemiological data extracted from patient medical charts included: patient age; patient sex; hospital; ward; whether the patient was a resident of a congregate setting; the number of hospitalizations in the past year; the principal admission diagnosis; the length of stay at time of specimen collection; whether the patient was receiving antibiotics at specimen collection; which antibiotics were being received at specimen collection (if any); which antibiotics were used to treat the infection; treatment duration; patient outcome; and whether hospital discharge summary included infection as a cause of death, if relevant.

The primary objective of the study was to determine, using whole-genome sequencing (WGS) combined with epidemiological data, whether clinically unrecognized clusters of transmission of *S. marcescens* are occurring in the hospital setting.

### Laboratory Methods

In the clinical microbiology laboratory, specimens were identified as per local standard operating procedures using conventional bacterial media and incubation conditions appropriate to the various specimen types submitted by requesting physicians. All isolates underwent final confirmatory identification using the VITEK MALDI TOF-MS (bioMérieux) using the V.3.2 database to assign species identifications, and antimicrobial susceptibility testing (AST) using the VITEK 2 system (bioMérieux), which performs miniaturized broth microdilution MIC testing using an automated Gram-negative AST-N390 card. MICs were interpreted by the VITEK 2 system according to the Clinical and Laboratory Standards Institute (CLSI) M100 guideline as used in the laboratory at the time of testing [18]. Bacterial isolates were frozen at −80°C in a 25% glycerol stock solution and transported for WGS.

We purified genomic DNA (gDNA) using the Wizard Genomic DNA Purification Kit (Promega) as per manufacturer instructions and subsequently quantified using the PicoGreen dsDNA Assay (ThermoFisher) to ensure a minimum gDNA concentration of 30 ng/µL was available for downstream applications. Individually barcoded short-read libraries were enriched for approximately 1000 bp insert sizes using the NEBNext Ultra II FS DNA Library Prep Kit (NEB) and the ProNex Size-Selective Purification System (Promega). The library was sequenced on the Illumina NextSeq 2000 to generate 2 × 150-bp paired-end reads from an average of 3.2 million clusters (range, 0.6–5.6 million).

### Bioinformatics

To filter and trim adapter sequences from the raw reads, we used Trimmomatic version 0.38 [19] specifying a quality score cutoff of 3 and a minimum read length of 36 base pairs. We then assembled filtered reads using Unicycler version 0.5.1 [20]. We evaluated genome assembly quality and calculated whole-genome guanine-cytosine content using Quast version 5.2.0 [21]. To annotate contigs to identify relevant genes, we used Bakta version 1.5.0 [22]. We then queried the Comprehensive Antibiotic Resistance Database (CARD, version 3.3.0) Resistance Gene Identifier (RGI) tool [23, 24] to predict the resistomes of patient isolates from the assembled contigs using the -include nudge argument in rgi main. RGI version 5.2.1 was used to summarize the output using rgi heatmap.

We used the Genome Database Taxonomy (GTDB)-Tk software version 2.4.0 [25] to assign each genome to a domain-specific reference tree and subsequently use each genome's relative evolutionary divergence and average nucleotide identity to determine its taxonomic assignment. We inferred a maximum-likelihood phylogenetic tree using IQ-TREE version 2.2.6 [26] and visualized phylogenetic relationships using iToL version 7 [27]. We used the Solu cloud-based platform version 1.0.251 [28] to run MOB-Suite version 3.1.9 [29] for typing, reconstruction, and visualization of predicted plasmids.

### Analysis

We performed a retrospective chart review to extract the aforementioned patient and microbiological variables of interest. We used conventional epidemiology combined with WGS data to determine if isolates from different patients were spatiotemporally linked. If patients overlapped within 1 month and/or space (ie, admitted to same ward) and/or exposure to procedures and their *Serratia* isolates clustered together on visual inspection of the phylogenetic tree, we considered there to be a possible epidemiological link between patients. We then calculated pairwise average nucleotide identity (ANI) for possibly linked specimens using OrthoANIu [30]; we considered an ANI ≥ 99.0% to be significant. We used descriptive statistics to summarize the characteristics of patients and their *S. marcescens* complex isolates. We used command-line tools for genome annotation and R, version 4.4.2 (R Foundation for Statistical Computing) to calculate relevant statistics. We used the packages ggplot2 [31], dplyr [32], diagrammeR [33], and ComplexHeatmap [34] to create data visualizations.

### Sequencing Data

Whole-genome sequences for all *S. marcescens* complex isolates included in the study and their linked antimicrobial MIC data are publicly available under NCBI BioProject PRJNA1232785.

## RESULTS

**Figure 1.**
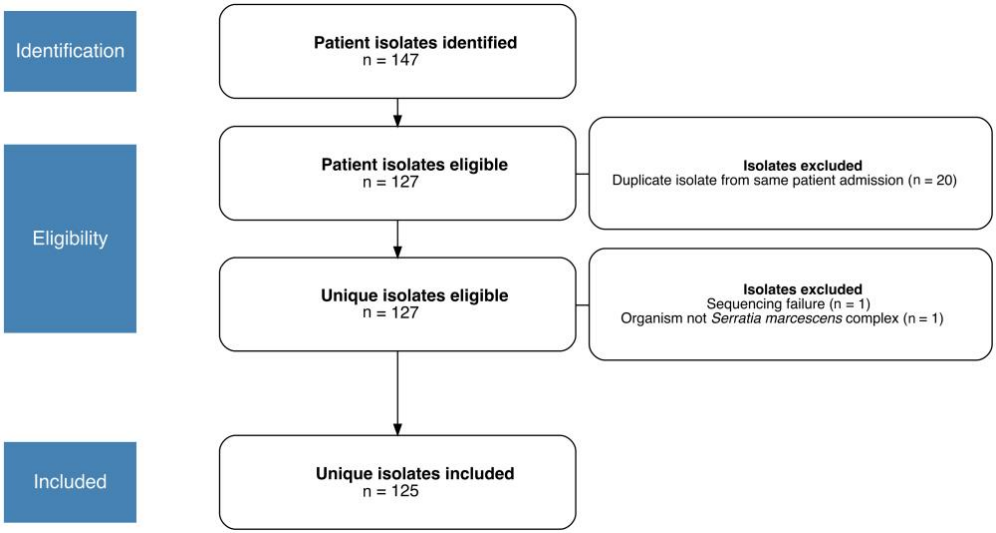
Participant flow chart and exclusion criteria.

We identified 147 microbial isolates as *S. marcescens* by our clinical microbiology laboratory during the study period. No suspected nosocomial *S. marcescens* complex outbreaks occurred during the study period at the participating institutions. Of these 147 isolates, 125 unique isolates were included in the analysis (Figure 1). Twenty-two patient isolates were excluded for the following reasons: 20 isolates were duplicates, 1 isolate failed WGS, and 1 sequenced isolate was identified as *Aeromonas caviae* (the initial patient specimen was identified as a mixture of *S. marcescens* and *A. caviae,* and the incorrect isolate was frozen for study inclusion). Most included patients were male (n = 88/125, 70.4%) and the majority were community-dwelling (n = 114/125, 90.5%). Patient demographic data are further described in Table 1. Of note, 49.6% (n = 62/125) of the included patients were receiving empiric antimicrobials at the time of specimen collection; however, 32.0% (n = 40/125) did not receive a definitive treatment course after cultures had been obtained, suggesting that these isolates were assessed by the treating clinician to be indicative of microbial colonization. Seventeen patients died during their hospital admission, with mortality attributable to *S. marcescens* complex infection in 64.7% (n = 11/17). For all individual patient-level demographic and microbial isolate data that were extracted as part of the study, including MICs for all tested antimicrobials, please see data freely accessible via the Open Science Framework [35].

**Table 1.** Demographics of Patients With *Serratia marcescens* Complex Infections

Patient Characteristic	All Included Patients (n *=* 125)	Patients Identified in 2022 (n = 71)	Patients Identified in 2024 (n *=* 54)
Age, y, median (IQR)	69.0 (57.0–76.0)	67.0 (59.5–76.0)	69.5 (51.5–78.5)
Female, n (%)	37 (29.6)	21 (29.6)	16 (29.6)
Congregate setting, n (%)	11 (8.9)	5 (7.0)	6 (11.1)
Median hospitalizations in past year, n (IQR)	0 (0–1.0)	1.0 (0–1.0)	0 (0–1.0)
Hospital length of stay at specimen collection, d, median (IQR)	1.0 (0–10.0)	1.0 (0–7.0)	2.0 (1.0–20.0)
Receipt of antimicrobials prior to specimen collection, n (%)	62 (49.6)	36 (50.7)	26 (48.1)
Receipt of carbapenem as empiric treatment, n (%)	5 (4.0)	4 (3.2)	1 (1.8)
Receipt of carbapenem as definitive treatment, n (%)	36 (28.8)	17 (23.9)	19 (35.2)
Treatment duration, d, median (IQR)	7.0 (7.0–14.0)	7.0 (7.0–14.0)	7.0 (7.0–10.7)
Patients not treated for positive culture result, n (%)	40 (32.0)	16 (22.5)	24 (44.4)
Patient deceased, n (%)	17 (13.6)	12 (16.9)	5 (9.3)
Of deceased, n (%) with infection as attributable cause of mortality	11 (64.7)	6 (50.0)	5 (100.0)

Abbreviation: IQR, interquartile range.

**Figure 2.**
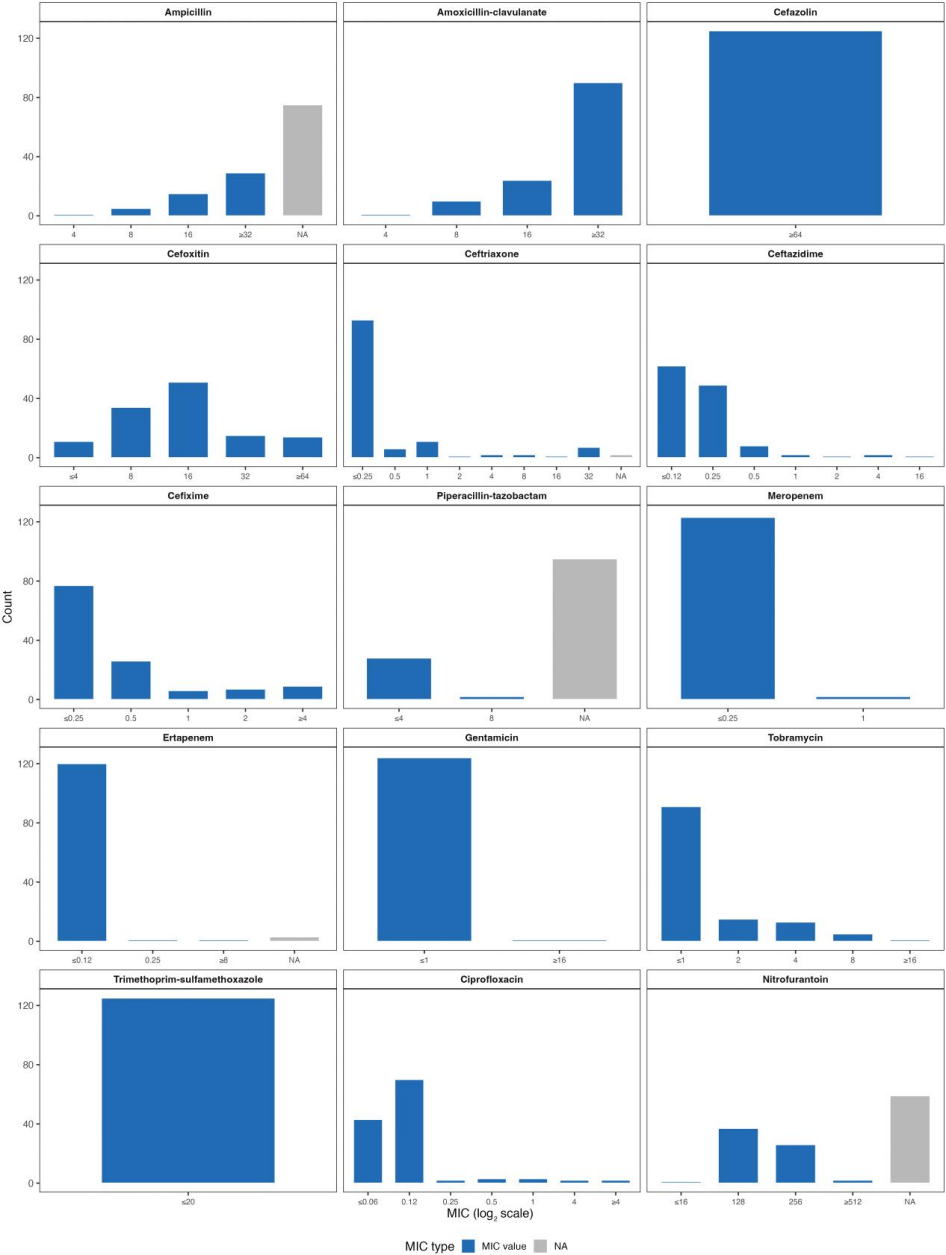
Distribution of antimicrobial MICs for *Serratia marcescens* complex isolates. Abbreviations: MIC, minimum inhibitory concentration; NA, not applicable.

**Figure 3.**
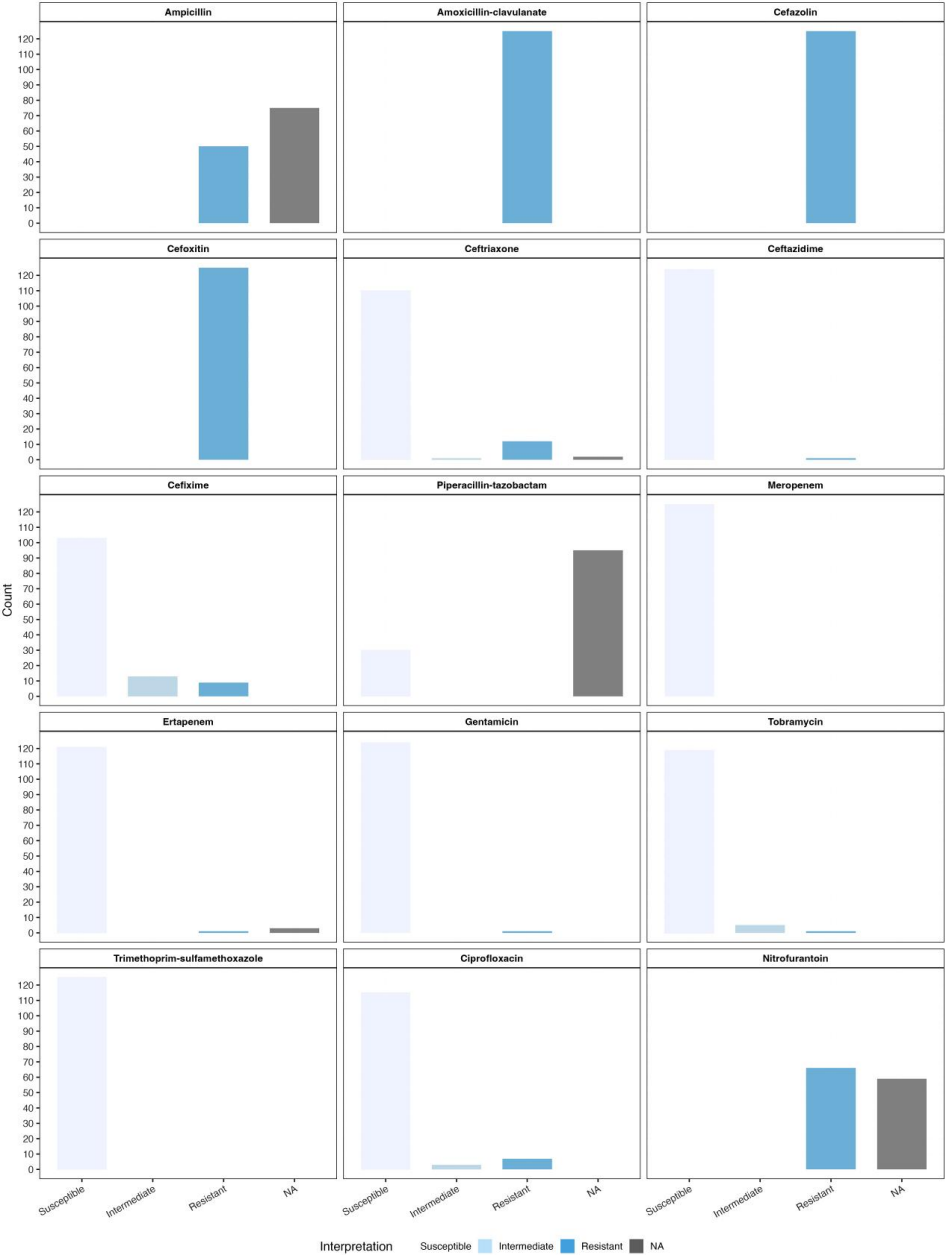
Distribution of antimicrobial interpretative breakpoints for *Serratia marcescens* complex isolates. Abbreviation: NA, not applicable.

We identified *S. marcescens* from various specimen types, including urines, tissues, fluids, swabs, and sputa (Table 2). All (n = 125/125) included microbial isolates were identified as *S. marcescens* by the clinical microbiology laboratory’s VITEK MALDI-TOF MS. AST using the VITEK 2 instrument demonstrated 2 major phenotypic patterns of AmpC expression in our clinical *S. marcescens* complex isolates: firstly, resistance to amoxicillin-clavulanate and cefoxitin, with susceptibility to ceftriaxone, suggestive of inducible AmpC expression; secondly, resistance to all 3 aforementioned agents, suggestive of derepression. Most isolates (n = 109/125, 87.2%) had an inducible AmpC expression, whereas only 9.6% (n = 12/125) exhibited a constitutive derepression phenotype using the definition of Aracil-Gisbert et al [8]. No included isolates exhibited a basal-like AmpC phenotype with susceptibility to all 3 agents. The distributions of MIC values and CLSI interpretative breakpoints for all tested antimicrobials are in Figure 2 and Figure 3.

**Table 2.** Microbiological Specimen Characteristics for Patient Isolates With *Serratia marcescens* Complex Infections

Microbiological Characteristic	All Included Patients (n *=* 125)
Specimen source, n (%)	
Bronchoalveolar lavage fluid	6 (4.8)
Blood	18 (14.4)
Body fluid	8 (6.4)
Sputum	22 (17.6)
Tissue	5 (4.0)
Urine	49 (39.2)
Wound swab	17 (13.6)
Species identification by MALDI, n (%)	
*Serratia marcescens*	125 (100.0)
Discordant species-level identification, MALDI vs WGS, n (%)	64 (51.2)
Species identification by NGS, n (%)	
*Serratia bockelmannii*	19 (15.2)
*Serratia marcescens* sensu stricto	61 (48.8)
*Serratia nematodiphila*	4 (3.2)
*Serratia nevei*	19 (15.2)
*Serratia ureilytica*	22 (17.6)
Antimicrobial susceptibility profile, n (%)	
Basal AmpC expression^a^	0 (0)
Inducible AmpC expression^b^	109 (87.2)
Derepressed AmpC expression^c^	12 (9.6)
Not applicable^d^	4 (3.2)

Abbreviations: MALDI, matrix-assisted laser desorption ionization time-of-flight mass spectrometry; WGS, whole-genome sequencing.

^a^Defined as testing susceptible to amoxicillin-clavulanate, cefoxitin, and a third-generation cephalosporin using the VITEK 2 antimicrobial susceptibility testing instrument.

^b^Defined as testing resistant to amoxicillin-clavulanate and cefoxitin, and susceptible to a third-generation cephalosporin using the VITEK 2 antimicrobial susceptibility testing instrument.

^c^Defined as testing resistant to amoxicillin-clavulanate, cefoxitin, and a third-generation cephalosporin using the VITEK 2 antimicrobial susceptibility testing instrument.

^d^In the event that 1 of the 3 antimicrobials used to predict AmpC resistance profile was not tested or tested intermediate, a value of “not applicable” was assigned.

We performed WGS on all 125 unique clinical isolates. The median genome size of the sequenced *S. marcescens* complex isolates was 5.10 Mb (range, 4.34–5.74 Mb) with a median guanine-cytosine content of 59.8% (range, 58.5%–60.3%). The median contig number was 36 (range, 17–1501), with a median largest contig length per genome of 760 kb (range, 38–2380 kb) and a median N50 statistic of 317 kb. The average sequencing coverage per base was 197-fold (range, 37–330).

When comparing the species-level identification between the VITEK MALDI-TOF MS used in the clinical laboratory and those assigned based on the whole-genome sequences using GTDB-Tk, we found roughly one-half (n = 64/125, 51.2%) to be discordant. Of the discordant identifications, all were found to be members of the *S. marcescens* complex, with 19 *S. bockelmannii,* 4 *S. nematodiphila,* 19 *S. nevei,* and 22 *S. ureilytica* isolates.

**Figure 4.**
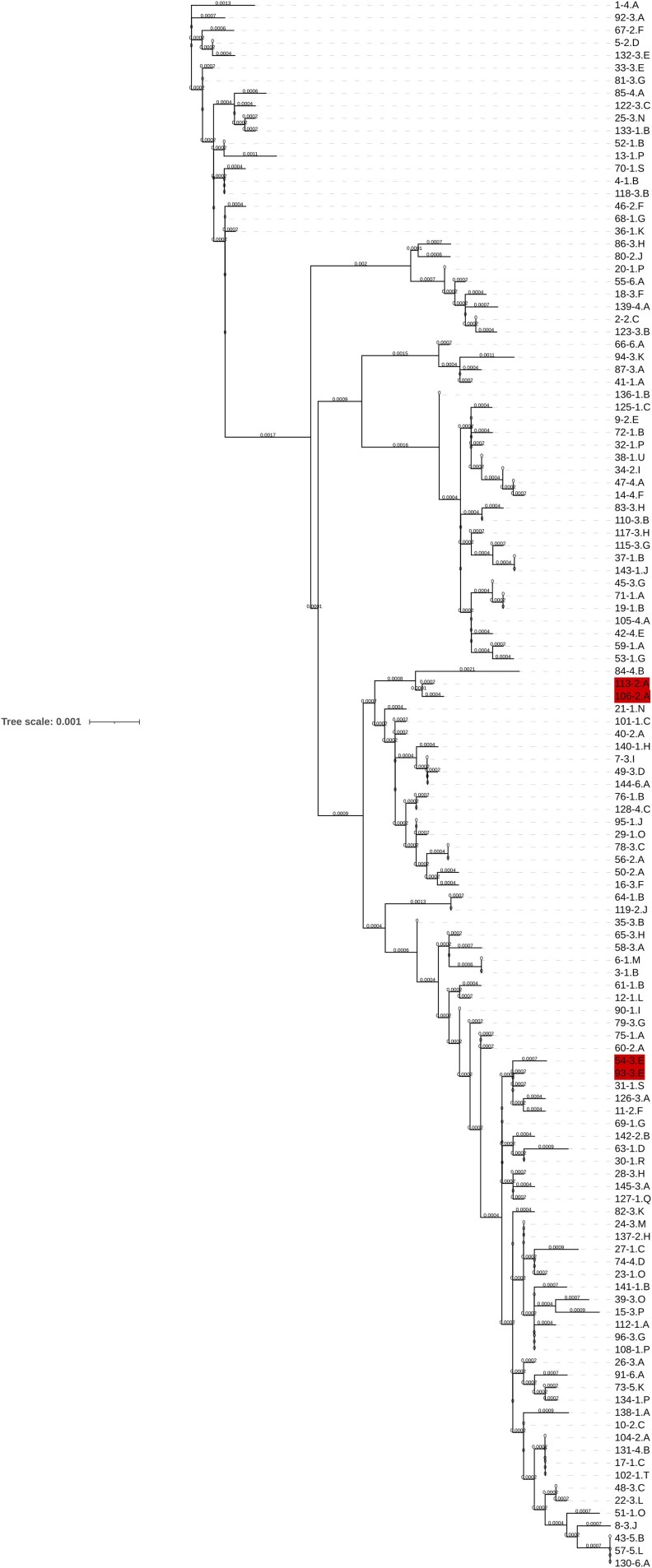
Phylogenetic tree of *Serratia marcescens* complex clinical isolates Each node on the phylogenetic tree has been assigned a unique identifier of the format “x-y.z”, where x is the numeric study identification number, y is the anonymized numeric code for the hospital where the specimen originated, and z is the anonymized letter code for the ward where the specimen originated. Adjacent nodes are labelled in red if patients had spatiotemporal overlap. Node lengths are labelled directly on the tree. Tree visualized using iToL, version 7 [27].

The inferred maximum-likelihood phylogenetic tree is shown in Figure 4. While many isolates clustered together phylogenetically (eg, isolates 43, 57, 130), most did not share a spatiotemporal link. In instances where isolates clustered together phylogenetically and were isolated from patients on the same hospital ward, we did not consider them putatively linked if there was no temporal overlap (eg, isolates 54 and 93) because the study did not include environmental sampling to help inform a putative link. However, 2 pairs of isolates appeared to cluster together phylogenetically and overlap spatiotemporally: isolate 50 and 56, and isolate 106 and 113.

For isolates 50 and 56, both of these *S. nevei* isolates were collected within 1 month of each other in July and August 2024, respectively, on postadmission day 1. We performed a pairwise USEARCH alignment using the OrthoANIu tool, which showed 98.80% ANI over an average aligned length of 3.74 Mb (isolate 50 genome coverage 70.41%, isolate 56 genome coverage 68.64%). This isolate pair did not therefore meet the prespecified ANI cutoff of ≥ 99.0%.

For isolates 106 and 113, both of these *S. nevei* isolates were collected in March 2024 within 10 days of each other, with each isolate collected on postadmission day 1. A pairwise USEARCH alignment performed using the OrthoANIu tool showed 99.27% ANI over an average aligned length of 3.77 Mb (isolate 106 genome coverage 75.53%, isolate 113 genome coverage 76.29%). A comparison of the phenotypic AST data for isolates 106 and 113 shows identical phenotypic MICs for all antimicrobials tested, apart from cefoxitin (isolate 106 MIC = 16 mg/L, isolate 113 MIC ≤4 mg/L) and cefixime (isolate 106 MIC = 0.5 mg/L, isolate 113 MIC ≤ 0.25 mg/L).

We identified 21 different predicted resistance determinants in the 125 sequenced isolates. The following genes were found in a majority of sequenced isolates: *emrR*, a fluoroquinolone efflux pump (n = 124/125, 99.2%); *PBP3*, a β-lactamase (n = 124/125, 99.2%); *adeF*, a fluoroquinolone and tetracycline efflux pump (n = 123/125, 98.4%); *ArnT,* a phosphoethanolamine transferase affecting peptide antimicrobials (n = 123/125, 98.4%); *CRP*, a macrolide, fluoroquinolone, and penam antimicrobial efflux pump (n = 123/125, 98.4%); *FosA8,* a fosfomycin thiol transferase (n = 123/125, 98.4%); the nitroimidazole efflux pump *msbA* (n = 123/125, 98.4%); *AAC(6′)-Ic,* which produces the aminoglycoside *N*-acetyltransferase enzyme (n = 122/125, 97.6%); *KpnF,* a multidrug efflux pump (n = 123/125, 98.4%); *qacG,* an antiseptic efflux pump (n = 122/125, 97.6%); *rsmA,* a fluoroquinolone, diaminopyrimidine, and phenicol antibiotic efflux pump (n = 122/125, 97.6%); *vanG,* which confers vancomycin resistance by altering peptidoglycan precursor structure (n = 121/125, 96.8%); *KpnH,* a multidrug efflux pump (n = 119/125, 95.2%); *SRT-2,* a β-lactamase (n = 90/125, 72.0%); and *tet*(*41*), a tetracycline efflux pump (n = 74/125, 59.2%).

**Figure 5.**
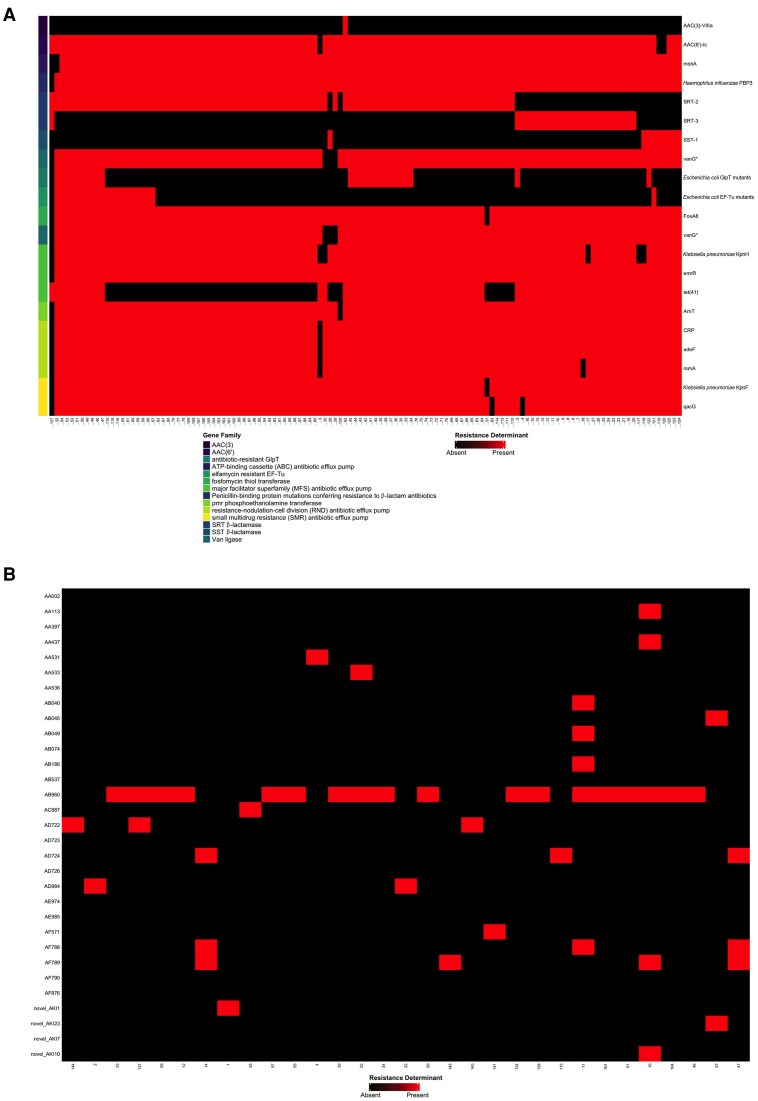
Heat map of comprehensive antimicrobial resistance database (CARD) resistance gene identifier (RGI) resistome prediction. *A*, Resistance determinants are mapped on the heat map as follows: the left column shows the gene family according to the CARD RGI ontology; the right axis labels show the gene names corresponding to the heatmap rows. The binary heat map shows either the presence of a resistance determinant for a given isolate in red, or the absence of said resistance determinant in black. The numerical isolate number is on the x-axis of the heatmap. *B*, Predicted plasmids are mapped on the heat map as follows: the y-axis shows the MOB type of the predicted plasmids; the x-axis labels show the numerical isolate number for those isolates that had predicted plasmids, only. The binary heat map shows either the presence of a predicted plasmid for a given isolate in red, or the absence of said resistance determinant in black.

A heat-map of CARD RGI predictions is found in Figure 5*A* for all included isolates. The majority of isolates (n = 114, 91.2%) harbored AAC(6′)-Ic, which inactivates aminoglycoside antibiotics; however, notably, only 1 isolate (0.8%) tested resistant to gentamicin and tobramycin phenotypically. Similarly, there was near-universal presence of multiple multidrug efflux pumps with fluoroquinolone targets; however, only 8 isolates (6.4%) demonstrated phenotypic resistance to ciprofloxacin. Of note, isolates 106 and 113 shared an identical predicted resistome (Figure 5*A*).

A heat-map of the isolates that had predicted plasmids is in Figure 5*B*. In total, 42 of 125 isolates (33.6%) harbored plasmids; among those with plasmids, the median plasmid number per isolate was 1 (IQR, 1–2). The most common plasmid identified belonged to MOB cluster AB960 and is a nonmobilizable *S. marcescens* plasmid occurring in 20 of 42 isolates (47.6%). We identified 4 novel plasmids (NCBI Biosample SAMN47405310, SAMN47405316, SAMN47405319, and SAMN47405332), of which 3 were found to be conjugative. Plasmid maps for the novel predicted plasmids are found on the Open Science Framework [35]. Notably, no predicted plasmids harbored AmpC operons or carbapenemases.

## DISCUSSION

This multiyear study of patients at 6 Canadian hospitals from 2022 to 2024, with invasive specimens identified as *S. marcescens* by the clinical microbiology laboratory sheds light on the epidemiology of *S. marcescens* complex infections in the hospital context. This study represents the largest known multiyear, multihospital survey of *S. marcescens* complex populations to use WGS in its analysis. We found significant genotypic and phenotypic heterogeneity amongst *S. marcescens* complex isolates, corroborating recent findings [7, 8, 36]. Our study confirms the presence and coexistence of multiple members of the *S. marcescens* complex in hospitalized patients. Our findings have significant implications for the laboratory diagnosis and clinical prevention of infections with *S. marcescens* complex organisms.

We found that VITEK MALDI-TOF MS provides poor species-level discrimination of *S. marcescens* complex isolates despite a high (≥ 95%) certainty of species-level identification in all included isolates (n *=* 125, 100%) when compared to the WGS taxonomic identification. The VITEK MALDI-TOF MS United States Food and Drug Administration 501(k)-cleared database, version 3.2 [37] does not contain *S. bockelmannii, S. nematodiphila, S. nevei,* or *S. ureilytica,* which explains the misidentification of multiple isolates as *S. marcescens*. This corroborates a recent report by Harch et al showing the similarly poor correlation of the Bruker MALDI-TOF MS platform's identification with WGS for *S. marcescens* complex [36]. The failure of multiple commercial MALDI-TOF MS platforms to accurately call *S. marcescens* complex isolates to the species level suggests that, while the clinical relevance of each species within *S. marcescens* complex is being further elucidated, laboratory reporting and MALDI-TOF MS databases should identify isolates only to the level of *S. marcescens* complex. This finding is likely due to the mass spectrum databases used in commercial MALDI-TOF MS being insufficiently granular: while the number of strains incorporated in these commercial databases is not publicly available, the current 501(k)-cleared bioMérieux database only includes *S. marcescens* amongst the *S. marcescens* complex. It may also have significant relevance for infection prevention and control efforts, as it may be necessary to rely on costlier WGS or multilocus sequence typing to characterize *S. marcescens* complex isolates in a suspected outbreak rather than the conventional clinical species-level identification provided by MALDI-TOF MS.

Interestingly, we found 1 pair of isolates over the study period that originated from the same hospital and ward within 10 days of each other, both sampled on postadmission day 1. While there were slight differences in phenotypic AST profiles, we note the isolates had an identical CARD RGI resistome prediction profile. Without concomitant environmental sampling, we are unable to establish a higher-certainty causal link. However, the presence of > 99.0% ANI and spatiotemporal linkage suggests that the hospital environment or patient-to-patient transmission may have contributed to, or played a clinically unrecognized role in, these particular infections. While 1 other isolate pair had spatiotemporal linkage, it did not have a sufficiently similar ANI value to be termed a putative transmission event. No further cases with spatiotemporal linkage or sufficiently similar ANI were identified over the remainder of the study period originating from the same hospital ward, suggesting that in-hospital transmission remains rare overall.

Contrary to the recent findings of Aracil-Gisbert and colleagues, we did not identify a subpopulation of *S. marcescens* complex organisms in our study displaying a basal AmpC expression profile with susceptibility to amoxicillin-clavulanate, cefoxitin, and a third-generation cephalosporin [8]. Our study population revealed susceptibility profiles consistent with inducible and derepressed AmpC expression only, although this difference may be explained by our lack of environmental isolates. It is possible that AmpC expression plays a role in adaptation to nonhost environments and altered gene expression may lead to a basal-type susceptibility pattern [8]. In line with current Infectious Diseases Society of America clinical guidelines denoting *S. marcescens* complex a low-risk AmpC-producing Enterobacterales [38], only 9.6% of our isolates had a susceptibility profile consistent with derepression. Our microbial isolates also lacked any predicted carbapenemases.

Our study has several limitations worth mentioning. Most notably, the methodology used cannot establish a causal link for pathogen spread between patients, particularly because our analysis did not include prospectively collected environmental samples because of its retrospective analytic nature. In addition, our study was powered for infection prevention and control-related outcomes (ie, transmission); therefore, no reliable conclusions can be made regarding clinical outcomes in this cohort, which would require a larger sample size. The retrospective chart review of patient data is prone to misclassification bias and is susceptible to confounding from unmeasured variables; we attempted to minimize this risk by using a prepiloted data extraction form and identifying consecutive patients over multiple time periods. Our analyses should thus be interpreted with caution and awareness of the context in which they were produced.

In conclusion, this study represents the largest known multiyear, multihospital survey of *S. marcescens* complex infections. Our work implies that current MALDI-TOF MS-based identification methods in the clinical laboratory insufficiently reflect the granularity of *S. marcescens* complex species constituents, which may have important implications for the investigation of (suspected) hospital outbreaks for infection control practitioners. Over our study period, we identified only 1 pair of *S. marcescens* complex isolates from distinct patients with a spatiotemporal link and a significant average nucleotide identity value. The role of environmental niches within the hospital system should continue to be elaborated upon in future research, to help inform infection prevention and control efforts for *S. marcescens* complex.
